# Triclosan exposure and in vitro fertilization treatment outcomes in women undergoing in vitro fertilization

**DOI:** 10.1007/s11356-020-11287-w

**Published:** 2020-10-23

**Authors:** Paweł Radwan, Bartosz Wielgomas, Michał Radwan, Rafał Krasiński, Anna Klimowska, Radosław Zajdel, Dorota Kaleta, Joanna Jurewicz

**Affiliations:** 1 Department of Gynecology and Reproduction, “Gameta” , 7 Cybernetyki St, 02-677 Warsaw, Poland; 2 Department of Gynecology and Reproduction, “Gameta” Kielce-Regional Science-Technology Centre, 45 Podzamcze St Chęciny, 26-060 Kielce, Poland; 3 Department of Toxicology, Medical University of Gdańsk, 107 Hallera St, Gdańsk, Poland; 4 Department of Gynecology and Reproduction, “Gameta” Hospital, 34/36 Rudzka St, 95-030 Rzgów, Poland; 5 Faculty of Health Sciences, Mazovian State University in Plock, 2 Dabrowskiego Sq, 09-402 Plock, Poland; 6 Chair of Business and Informatics, University of Łódź, 3/5 POW St., 90-255 Łódź, Poland; 7 Department of Hygiene and Epidemiology, Medical University of Lodz, Zeligowskiego 7/9 St, 90-752 Łódź, Poland

**Keywords:** Early IVF outcomes, Urinary triclosan concentrations, Environmental exposure, Implantation rate, Clinical pregnancy, Top quality embryo

## Abstract

Triclosan (TCS) is a widespread environmental endocrine-disrupting chemical. Animal and in vitro studies suggested that triclosan may affect homesostasis of sex and thyroid hormones and impact on reproduction. Due to limited data derived from human epidemiological studies, this study was performed to examine the association between urinary concentration of triclosan and in vitro reproductive outcomes (methaphase II (MII) oocyte yield, top quality embryo, fertilization rate, implantation rate, and clinical pregnancy) among women from infertility clinic. The study participants were enrolled in an Infertility Center in Poland. A total of 450 women aged 25–45 (*n* = 674 IVF cycles) provided urine samples. The urinary concentrations of triclosan were evaluated using validated gas chromatography ion-tap mass spectrometry method. Clinical outcomes of IVF treatment were abstracted from patients electronic chart records. Triclosan was detected in urine of 82% of women with geometric mean 2.56 ± 6.13 ng/mL. Urinary concentrations of triclosan were associated with decrease implantation rate (*p* = 0.03). There were no association between other examined IVF outcomes: MII oocytes, embryo quality, fertilization rate, and exposure to triclosan. As this is one of the first study on this topic, studies among larger and more diverse population are needed to confirm the results.

## Introduction

Man-made, widespread, environmental chemicals which have been used widely for decades have the ability to disrupt hormonal homeostasis and affect human fertility. One of this chemicals is triclosan (TCS) used in personal care products such as soaps and toothpaste, household products, and pharmaceuticals as broad-spectrum antibacterial additives (Yuan et al. 2015). Because of widespread usage of triclosan, there is the potential for the general population to be exposed to triclosan.

Environmental exposure to triclosan may occur via consumer products that contain TCS, but also through water and/or food products contaminated with TCS (Weatherly and Gosse 2017). Detectable levels of TCS were found in milk and blood of nursing mothers (0.25–2.1 μg/L) (Allmyr et al. 2006) and human urine (2.4–3.7 μg/L) (Calafat et al. 2008). After oral exposure, urinary excretion increases in humans within 24 h. During the first 4 days after exposure, between 24 and 83% of the consumed TCS is excreted, and after 8 days, excretion approaches baseline levels (Sandborgh-Englund et al. 2006).

Triclosan has been reported to act as endocrine disrupting chemical in many in vitro, animal, and also human studies (Axelstad et al. 2013; Louis et al. 2017). Recent animal studies correlated TCS exposure with adverse reproductive effects. Exposure to TCS on certain gestational days decreased implantation rates in mice (Crawford and Decatanzaro 2012). In the study performed by Hwang et al. (2014), embryos are treated with higher than 1.0 μM levels of TCS displayed arrested development. Human studies on potential effect on reproduction and fertility, especially early IVF outcomes are limited and inconclusive. Lange et al. (2015) found that TCS concentrations in urine decrease oocyte yield. Other examined clinical IVF outcomes (implantation rate, pregnancy rate, or live birth rate) were not related to such exposure (Lange et al. 2015). Recently, published study by Hua et al. (2017) found the negative effect of TCS exposure on top quality embryo and implantation rate (Hua et al. 2017).

The aim of the study was to investigate the effect of environmental exposure to triclosan and early reproductive outcomes (MII oocyte count, top quality embryo, fertilization rate, implantation rate, and clinical pregnancy). According to our best knowledge, the current study is the largest human epidemiological study addressing the topic of triclosan exposure and early adverse reproductive outcomes.

## Materials and methods

### Study participants and data collection

A total of 450 women between 25 and 45 years of age seeking infertility treatment at Gameta Hospital reproductive centre certified by the European Society for Human Reproduction and Embryology (ESHRE ART Center Certification for good clinical practice, 2019, C-0001) and that underwent at least one fresh in vitro fertilization cycle (*n* = 674 IVF-ICSI cycles) were recruited. The couples’ exclusion criteria were as follows: fertilization failure during the previous IVF-ICSI attempt, sperm concentration < 1 million per mL, azoopsermia, ovarian hyperstimulation syndrome, abnormal pelvic ultrasound examination (abnormal uterine cavity, hydrosalpinx, ovarian cysts), endocrinologic disorders (POCS (polycystic ovary syndrome)), menstrual disorders, chlamydia infection, thyroid disfunction (TSH > 2.5 μU/mL, BMI > 40 kg/m^2^). The Bioethical Committee in Lodz, Poland, approved the study (resolution no 23/2014). At the time of recruitment, study subjects received written informed consents before their participation and completed a questionnaire about socio-demographic characteristics, medical, especially gynecological history, chronic diseases, lifestyle factors, and occupational factors. The participant’s date of birth was collected at entry, and weight and height were measured by trained study staff. Body mass index (BMI) was calculated as weight (in kilograms) divided by height (in meters) squared.

### Clinical data assessment

Participants’ clinical data were received from the medical electronic charts record. Concentration of hormones: follicle-stimulating hormone (FSH), estradiol (E2), luteinizing hormone (LH), and progesterone, were assessed in serum using chemiluminescence immunoassay between second and third day of menstrual cycle. Serum was analyzed for anti-müllerian hormone (AMH) with an enzyme-linked immunoabsorbent method utilizing commercially available Gen-II ELISA kits according to manufacturer instruction (Beckman Coulter, Inc., USA). The highest level of oestradiol prior to oocyte retrieval was treated as the peak oestradiol level.

#### Ovarian stimulation and ovum pick up

The long agonist or short antagonist protocol was used to stimulate ovulation. The administrated drugs were recombinant FSH (rFSH) or human menopausal gonadotropin (hMG) at a daily dose of 150–300 IU. In long protocol**,** patients daily received GnRH agonist (0.1 mg Gonapeptyl, Ferring Pharmaceuticals) and transvaginal ultrasound examination and measuring the serum level of estradiol (E2) w**ere** used to monitor the growth of follicles. In short antagonist protocol**,** the ovarian stimulation was started on the second day of the cycle. The patients were administered 0.25 mg of Ganirelix (Orgalutran, Organon) when mean diameter one of the follicles was bigger than 14 mm or when the estradiol level was above 400 pg/m**L**. After the diameter of the follicles was above 17 mm and the estradiol level was above 200 pg/m**L** per one follicle, the patient was administered subcutaneously 250 μg rhCG (Ovitrelle, Merck-Serono) or 0.2 mg triptorelin (0.1 Gonapeptyl Dailly, Ferring)**.** Ovarian pick up (OPU) was preformed general intravenous anesthesia 36 h following the injection of rhCG/GnRH analogue. In order to supplement the luteal phase**,** the patients were intravaginally administered 2 × 200 mg micronized progesterone (Luteina, Adamed, Poland) and 3 × 10 mg oral **d**ydrogesteron (Duphaston, Mylan Healthcare, Poland). In case of an increased risk of ovarian hyperstimulation syndrome (OHSS, defined as at least, 14 follicles of ≥ 11 mm**)** or an elevated progesterone concentration (> 1.5 ng/ml) on day of final follicle maturation**,** a “freeze all” strategy was employed.

#### Oocyte preparation

Retrieved follicular fluid was examined under a microscope to identify and isolate cumulus-oocyte complexes (COC). Once retrieved, COC were placed in G-IVF PLUS (Vitrolife, Sweden) and preincubated for 2 h at 37 °C, 6% CO_2_, 5% O_2_ before denudation. Granulosa cells from COC were removed in two-step procedure: initial enzymatic digestion in a solution of 80 IU/mL hyaluronidase (Vitrolife, Sweden) followed by mechanical removal in G-MOPS PLUS (Vitrolife, Sweden). Quality and maturity of each oocyte were assessed under an inverted microscope. Oocytes were classified as follows: germinal vesicle (GV), metaphase I (MI), metaphase II (MII), or degenerated. The oocytes which achieved the metaphase of the second meiotic division (MII) were incubated in G-1 PLUS (Vitrolife, Sweden) for 1–2 h before fertilization.

#### Sperm preparation

Sperm preparation was proceeded by centrifugation in QUINN’s Sperm Washing Medium (SAGE, USA) at 250 g for 10 min. Before the ICSI procedure, the spermatozoons were immobilized in the drop of 7% PVP (Polyvinylpyrrolidone, SAGE, USA). Intracytoplasmic sperm injection was performed for all patients with a standard micromanipulators (Narishige, Japan) and inverted microscope (Nikon, Japan/Leica, Germany). Spermatozoa was microinjected with a × 200 magnitude in a drop of G-MOPS PLUS (Vitrolife, Sweden). After ICSI, oocytes were allocated into 500 μL of G-1 PLUS culture medium (Vitrolife, Sweden) in four-well dishes (Nunc, USA) and cultured at 37 °C, 6% CO_2_, 5% O_2_. In day 3, G-1 PLUS culture medium was replaced with G-2 PLUS (Vitrolife, Sweden).

#### Oocyte fertilization

A**n i**ntracytoplasmic sperm injection was performed for all patients with a standard micromanipulators (Narishige, Japan) and inverted microscope (Narishige, Nikon, Japan). Spermatozoa was microinjected with a **×** 200 magnitude in a drop of G-MOPS (Vitrolife, Sweden). Fertilized oocytes were placed in four-well dishes (Nunc, US**A**) in 500 μ**L** G-1 PLUS (group Vitrolife). Zygotes and embryos were cultured in sequential medium (G-1 P**LUS**/G-2 P**LUS**, Vitrolife, Sweden). The culture was conducted in standard conditions (6% CO_2_ and 5% O_2_, 37 °C) using HeraCell 150 incubators (Thermoscientific, Germany).

#### Embryo assessment

Zygotes were assessed on the basis of two pronuclei and two polar bodies in the perivitelline space 16–18 h following the application of the IVF-ICSI procedure. In days 2 and 3 (respectively, 43–45 and 67–69 h following the microinjection), the embryos were evaluated in terms of the number, size, symmetry of blastomers, multinucleation, and degree of fragmentation (grade A, B, C, D). Top quality embryos were defined as grade A and B in day 3. Blastocysts in day 5 and day 6 (114–118 and 138–142 h following the IVF-ICSI procedure) were assessed on the base of the classification of the blastocoels and the degree of embryonic expansion. The embryoblast and trophoblast were scored in expanded blastocyst according to modified Gardner scoring system (Gardner et al. 2000).

The percentage of embryos with successful implantation compared to the number of embryos transferred was treated as the implantation rate. Clinical pregnancy was recognized when β-hCG level increased and the confirmation of an intrauterine pregnancy on an ultrasound at 6 weeks.

### Assessment of urinary triclosan concentrations

Urine samples were collected in a polypropylene cup. During each IVF-ICSI cycle, at least one urine sample was provided just before egg-retrieved procedure. A handheld refractometer was used to assessed specific gravity (SG). The concentration of triclosan was measured using gas chromatography (Varian GC-450) coupled with tandem mass spectrometry (Varian 220-MS, ion-trap mass spectrometer as previously described (Jurewicz et al. 2019)). External quality control was carried out by participation in the German External Quality Control Scheme (G-EQUAS), organized, and managed by the Institute and the Outpatient Clinic for Occupational, Social, and Environmental Medicine of the University of Erlangen-Nuremberg (Erlangen, Germany). Scheme, evaluation, and certification are based on the German Federal Medical Council (http://www.g-equas.de/). Analytical laboratory of the Department of Toxicology, Medical University of Gdańsk, successfully participated in this external quality check for triclosan.

### Statistical analysis

Demographic, baseline, and clinical characteristics of the study participants are presented using mean ± standard deviations (SD) or percentages. Urinary triclosan concentrations were categorized into quartiles. The lowest quartile (< LOD to 25th) was considered as the reference group. Other categories of exposure were as follows: > 25th to 50th, > 50th to 75th, > 75th. Additionally, triclosan exposure was presented as continuous variable.

The levels of triclosan < LOD were assigned as half of LOD (Hornung and Reed 1990) and adjusted for urine dilution by specific gravity (SG) using the following formula: Pc = P[(1.016–1)/(SG-1)], where Pc is the SG-corrected triclosan concentration (μg/L), P is the measured triclosan concentration, and 1.016 is treated as the median SG level among study participants ((Hornung and Reed 1990). The triclosan concentrations were natural-log transformed (*x* + 1) to normalize distributions.

To explore the effect of triclosan exposure on early reproductive outcomes, multivariable generalized linear mixed analyses with random intercepts were performed. The variables such as age, BMI, smoking status, and infertility diagnosis were treated as the confounding factors. The level of statistical significance was set at 0.05. All statistical analysis were performed using R statistical software (ver.3) (R Core Team 2016).

## Results

Table 1 presents the demographic and medical characteristics of the study population. The mean age and BMI of participants were 31 years of age and 23.19 kg/m^2^, respectively. A total of 65.11% and 32.00% of women had higher or secondary education, respectively. Ninety-two percent of study participants declared not smoking cigarettes. A total of 54.44% of women drank none or less than 1 drink per week. In case of the infertility diagnosis, it was male factor in 38%, idiopathic infertility in 30.89%, and female factor in 28.67% (Table 1).

**Table 1:** Baseline characteristics and IVF outcome among the study population *N* = 450

Variables	*n* (%)	Mean ± SD
Education		
Vocational Secondary Higher	13 (2.89) 144 (32.00) 293 (65.11)	
Age (years)		31.28 ± 3.52
24–30 31–39 40–44	81 (18.00) 342 (76.00) 27 (6.00)	
BMI (kg/m^2^)		23.19 ± 2.67
< 18.5 18.5–24.9 25–29.9 30–40	26 (5.78) 261 (58.00) 135 (30.00) 28 (6.22)	
Current smoking		
No Yes	414 (92.00) 36 (8.00)	
Alcohol use		
None or < 1 drink/week	245 (54.44)	
1–3 drinks/week Everyday	198 (44.0) 7 (1.56)	
Initial infertility diagnosis		
Male factor Idiopathic Endometriosis Ovarian factor Tubal factor Missing data	171 (38.0) 139 (30.89) 62 (13.78) 21 (4.67) 46 (10.22) 11 (2.44)	
Duration of couple’s infertility (years)		
1–2 2–3 3–5 > 5	34 (7.56) 121 (26.89) 131 (29.11) 164 (36.44)	
Stimulation protocol		
Long GnRH agonist protocol GnRH-antagonist protocol	198 (44) 252 (56)	
AFC (*n*)		12.54 ± 7.21
FSH (IU/L) (mean ± SD)		6.21 ± 1.13
E2 peak (ng/mL)		2608.74 ± 1614.63
E2 (pg/mL)		92.78 ± 15.78
Progesterone (ng/mL)		0.99 ± 1.58
LH (IU/L)		5.34 ± 3.24
AMH (ng/mL)		1.19 ± 1.22
Number of oocytes retrieved (COC)		13.21 ± 7.44
Number of MII oocytes		9.98 ± 7.22
Fertilization rate (%)		80 ± 21
No. of cleavage embryos available		6.28 ± 1.54
No. of top quality embryos		2.01 ± 2.48
No. of embryos transferred		1.95 ± 0.16
Clinical pregnancy rate (%) per cycle		43.12 ± 7.89
Implantation rate (%) per embryo		38.16 ± 2.79

*SD*, standard deviation; *AMH*, anti-Müllerian hormone; *AFC*, antral follicle count; *FSH*, follicle-stimulating hormone; *E2*, estradiol; *LH*, luteinizing hormone

The antral follicle count was 12.54 ± 7.21 in both ovaries. The mean level of FSH, E2, LH, and progesterone was 6.21 ± 1.13 IU/L, 92.78 ± 15.78 pg/mL, 5.34 ± 3.24 IU/L, and 0.99 ± 1.58 ng/mL, respectively (Table 1). The peak estradiol level was 2608.74 ± 1614.63 ng/mL (Table 1).

The characteristic of early IVF outcomes is included in Table 1. The mean ± SD number of oocytes retrieved, MII oocytes, embryos available, top quality embryos, and embryo transferred was 9.98 ± 7.22, 13.21 ± 7.44, 6.28 ± 1.54, 2.01 ± 2.48, and 1.95 ± 0.16, respectively. The fertilization rate and implantation rate was 80 ± 21% and 38.16 ± 2.79%, respectively. The rate of clinical pregnancy was 43.12 ± 7.89 (Table 1).

As shown in Table 2, triclosan was detected in 81.22%. The geometric mean of TCS and TCS SG-adjusted of the 562 samples provided by 450 women was 2.56 ng/mL and 2.87 ng/mL, respectively.

**Table 2:** Urinary triclosan levels among study population

TCS	A mean ± SD	G mean ± SD	Min	Q25	Median	Q75	Q95	Max	> LOD (%)
Triclosan (ng/mL) (in = 450)	42.21 ± 139.55	2.56 ± 6.13	0.3	0.52	1.37	2.55	5.89	265.17	81.22
Triclosan (ng/mL) SG adjusted (*n* = 450)	37.12 ± 158.66	2.87 ± 6.12	0.3	0.77	1.42	2.67	6.23	278.78	81.22

*A mean*, aritmetic mean; *G mean*, geometric mean; *SD*, standard deviation; *Min*, minimal value; *Max*, maximum value; *Q25*, 25 percentile; *Q75*, 75 percentile; *Q95*, 95 percentile

The associations between exposure to triclosan and early reproductive outcomes are presented in Table 3. In the first regression model adjusted for BMI, age, smoking, and infertility diagnosis, the significant association was observed between triclosan concentration (continuous variable) and implantation rate (*p* = 0.047). In the fourth quartile of TCS, exposure decrease in implantation rate was also observed (*p* = 0.03). No significant associations were observed between exposure to triclosan and MII oocyte count, embryo quality, fertilization rate, and clinical pregnancy (Table 3). When the model was additionally adjusted for AFC and protocol type, the results were similar. Triclosan negatively affects the implantation rate in the fourth quartile of exposure (*p* = 0.03) and as continuous variable (*p* = 0.04) (Table 3).

**Table 3:** The association between triclosan level in urine and IVF outcomes

		MII oocyte count			Top quality embryo			Fertilization rate			Implantation			Clinical pregnancy		
		Coef	95% CI	*p*	Coef	95% CI	*p*	Coef	95% CI	*p*	Coef	95% CI	*p*	Coef	95% CI	*p*
TCS	Cont^a^	0.04	− 0.06; 0.27	0.75	− 0.06	− 0.09; 0.13	0.41	0.05	− 0.06; 0.25	0.66	− 0.19	− 0.40; − 0.01	*0.047*	− 0.01	− 0.06; 0.22	0.94
	Cont^b^	0.03	− 0.05; 0.30	0.77	− 0.04	− 0.06; 0.23	0.57	0.04	− 0.04; 0.12	0.52	− 0.17	− 0.38; − 0.02	*0.04*	− 0.01	− 0.05; 0.25	0.85
	Q2^a^	0.03	− 0.06; 0.27	0.82	− 0.19	− 0.43; 0.06	0.13	− 0.02	− 0.06; 0.09	0.28	− 0.01	− 0.06; 0.22	0.12	− 0.04	− 0.06; 0.12	0.70
	Q2^b^	0.03	− 0.04; 0.32	0.81	− 0.12	− 0.22; 0.04	0.09	− 0.02	− 0.04; 0.12	0.56	− 0.02	− 0.04; 0.28	0.14	− 0.03	− 0.05; 0.15	0.71
	Q3^a^	0.05	− 0.06; 0.32	0.72	− 0.03	− 0.06; 0.18	0.77	− 0.05	− 0.06; 0.17	0.68	− 0.11	− 0.16; 0.11	0.34	− 0.01	− 0.06; 0.04	0.12
	Q3^b^	0.04	− 0.04;0.28	0.73	− 0.04	− 0.05; 0.22	0.82	− 0.04	− 0.05; 0.36	0.78	− 0.12	− 0.15; 0.12	0.35	− 0.01	− 0.05; 0.07	0.14
	Q4^a^	0.10	− 0.06; 0.33	0.38	− 0.08	− 0.09; 0.16	0.50	− 0.05	− 0.06; 0.28	0.65	− 0.04	− 0.06; − 0.03	*0.03*	− 0.05	− 0.06; 0.17	0.65
	Q4^b^	0.11	− 0.04; 0.30	0.40	− 0.06	− 0.07; 0.19	0.56	− 0.04	− 0.05; 0.32	0.67	− 0.03	− 0.04; − 0.004	*0.03*	− 0.02	− 0.03; 0.18	0.59

*TCS*, triclosan; *Q2*, (25–50) percentile (*n* = 95); *Q3*, (50–75) percentile (*n* = 62); *Q4*, > 75 percentile (*n* = 96); reference category to Q2, Q3, and Q4 is Q1 Q1–≤ 25 percentile (*n* = 112); models adjusted for (a) BMI, age, smoking, and infertility diagnosis, (b) BMI, age, smoking, infertility diagnosis, protocol type, and AFC

## Discussion

In the current study, we assessed the exposure to triclosan and early in vitro reproductive treatment outcomes among women from infertility clinic. Urinary triclosan concentration decrease implantation rate, whereas no association was found between other examined parameters of early IVF outcomes: MII oocyte count, top quality embryos, fertilization rate, and clinical pregnancy.

As the studies on exposure to triclosan and fertility, especially early IVF outcomes, remain unclear and limited, the comparison of the results is difficult. Only two human epidemiological studies evaluate the urinary triclosan concentrations and IVF outcomes (Lange et al. 2015; Hua et al. 2017). Hua et al. (2017) observed that urinary TCS concentrations were negatively associated with top quality embryos and implantation rate among women undergoing in vitro fertilization, which is in line with current study. On the other hand, Lang et al. (2015) found a negative association between TCS exposure in second, third, and fourth quartile of exposure and number of oocyte retrieved. There were no significant differences with other examined clinical IVF outcomes (implantation, pregnancy rate, live birth). The differences in the results may arise from the fact that the study was based on smaller sample size (*n* = 134, 181 cycles) compared to our study (*n* = 450, 674 cycles) and the differences in women characteristics so the confounding factors although the same may differ. Additionally, the sensitivity of female reproductive track to TCS exposure may vary depending of the widow of exposure, when the exposure occurred to cause the adverse effect.

Other studies performed on TCS exposure have assessed fecundity. In the study performed among Canadian women, urinary triclosan concentration decrease fecundity (Vélez et al. 2015). Whereas in prospective cohort study among 501 couples, no association was found between triclosan exposure and time to pregnancy and couples’ fecundity (Smarr et al. 2017).

Although bisphenol A (BPA) and triclosan (TCS) are EDC with similar chemical structures to 17β-estradiol (Wolstenholme et al. 2011), many studies investigated the effect of exposure to BPA on fertility, whereas the TCS exposure on human reproduction, especially fertility, remains unclear and limited. Previously published studies suggest that triclosan may caused changes in estrogen levels, which can lead to implantation failure in humans and animals (Ma et al. 2003; Gidley-Baird et al. 1986) due to their ability to mimic the estrogen concentrations (Stoker et al. 2010; Ishibashi et al. 2004). Crawford and Decatanzaro (2012) found decrease in implantation rates and increase gestational length in mice after exposure to triclosan. Other studies assess the exposure to BPA. Xiao et al. (2011) observed reduction of implantation rates by affecting uterine receptivity, embryo transport, and development. Berger et al. (2007) and Takai et al. (2000) also reported reduce implantation sites in animal.

Urinary levels of triclosan in our study were lower than those reported in for female US population in similar age in 2014 (CDC 2017). The median, 75 percentile, and geometric mean were 1.37 ng/mL, 2.55 ng/mL, and 2.56 ng/mL, respectively, in our study and among US females (6.20 ng/mL, 31.7 ng/mL, 9.63 ng/mL, respectively). On the other hand, the TCS concentrations were higher than that presented by Hua et al. (2017) among infertile patients undergoing their first IVF/ICSI in China (0.058 ng/mL, 0.064 ng/mL, 0.645 ng/mL).

To the best of our knowledge, the current study is the largest human study (*n* = 450 women) that explore the effect of triclosan exposure and early reproductive outcomes.

Strengths of our study include performing study in the same center, using the same standardized protocol. Detailed questionnaire data on demographics, medical, and lifestyle risk factors allowed for control of confounding in the statistical analysis. Also, all study participants provided at least 1 urine sample per cycle which in case of nonperistant chemical with short half-lives is very important to confirm the exposure. Our study has also several limitations. It may be difficult to generalize the results to the general population as the study was conducted among women seeking infertility treatment. Additionally, the study is not able to show the mechanism of the observed associations, but the epidemiological study in their nature is not designed to study the mechanisms.

In conclusion, we found that urinary triclosan concentrations may affect early IVF outcome: implantation rate. As this is one of the first study, the confirmation of findings in larger studies is needed.

## Data Availability

The datasets used and analyzed during the current study are available from the corresponding author on reasonable request.
